# Association of circulating minerals and vitamins with pregnancy complications: a Mendelian randomization study

**DOI:** 10.3389/fnut.2024.1334974

**Published:** 2024-06-18

**Authors:** Yuan Xie, Jie Zhang, Shuang Ni, Ji Li

**Affiliations:** ^1^Department of Gynecology, Longhua Hospital, Shanghai University of Traditional Chinese Medicine, Shanghai, China; ^2^Central Laboratory for Research, Longhua Hospital, Shanghai University of Traditional Chinese Medicine, Shanghai, China

**Keywords:** vitamins, micronutrients, pregnancy complications, Mendelian randomization, causality

## Abstract

**Background:**

Though considerable studies suggesting connections between micronutrients and pregnancy complications, current evidence remains inconsistent and lacks causative confirmation. Our study aimed to explore the causal links between them with a two-sample Mendelian randomization (MR) analysis.

**Methods:**

Genome-wide association studies (GWAS) data for circulating micronutrients were sourced from GWAS Catalog consortium and PubMed, while data for pregnancy outcomes, including gestational diabetes mellitus (GDM), gestational hypertension (GH), spontaneous abortion (SA), preterm birth (PTB), and stillbirth (SB), were retrieved from the UK Biobank and FinnGen consortia. Causal effects were appraised using inverse variance weighted (IVW), weighted median (WM), and MR-Egger, followed by sensitivity analyses and meta-analysis for validation.

**Results:**

Genetically predicted higher vitamin E (OR = 0.993, 95% CI 0.987–0.998; *p* = 0.005) levels were inversely associated with SA risk. Consistent results were obtained in meta-analysis (OR = 0.99, 95% CI 0.99–1.00; *p* = 0.005). Besides, a potential positive causality between genetic predisposition to vitamin B12 and SB was identified in both IVW (OR = 0.974, 95% CI 0.953–0.996; *p* = 0.018) and WM analysis (OR = 0.965, 95% CI 0.939–0.993; *p* = 0.013). However, no causal relationships were observed between other analyzed circulating micronutrients and pregnancy complications.

**Conclusion:**

This study offers compelling evidence of causal associations between circulating levels of vitamins E, B12 and the risk of SA and SB, respectively. These findings are pivotal for pregnancy complications screening and prevention, potentially guiding clinical practice and public health policies toward targeted nutritional interventions.

## 1 Introduction

Gestational diabetes mellitus (GDM), gestational hypertension (GH), miscarriage, preterm birth (PTB), and stillbirth (SB) are prevalent complications encountered during pregnancy. GDM is notably the most common, affecting approximately 16.7% of live births (1). GH, which impacts 2–8% of pregnancies worldwide, is a primary cause of maternal and perinatal mortality (2). The incidence rates of miscarriage and preterm labor are reported to be 10.8% and 5–18%, respectively (3, 4). It is estimated that over 7,000 women globally experience stillbirth daily (5). As a result, pregnancy complications represent a substantial challenge to global maternal and neonatal health, highlighting the importance of early screening and prevention.

Although the precise cause of pregnancy complications remains elusive, a growing body of observational studies suggests that micronutrients, especially minerals and vitamins, might play a role in their onset (6–9). However, existing literature on the relationship between micronutrients and pregnancy complications provides mixed results, complicating the development of clear nutrient supplementation guidelines. For instance, a meta-analysis found that vitamin B12 deficiency was associated with an increased risk of PTB (10), but a randomization controlled trial (RCT) revealed no significant clinical benefit from multivitamin supplementation (7). Large cohort studies have indicated that increased folate intake from supplements is associated with a decreased risk of spontaneous abortion (SA) (11). In contrast, another prospective cohort study found vitamins had limited impact on the risk of early pregnancy loss or birth weight (12). These varying findings might be attributed to intrinsic limitations of observational studies, such as reverse causality and confounders, or to challenges in RCTs, such as adherence, dosage, trial duration, and statistical power. Thus, the definitive role of micronutrients in pregnancy complications remains to be determined.

Mendelian randomization (MR) has emerged as a method to infer causal associations between risk factors and health outcomes (13). Using the random assignment of genetic variants during meiosis, MR employs these genetic variations as instrumental variables (IVs) to explore associations between exposures and outcomes (14). As these genetic markers are predetermined at conception, prior to disease onset, MR analyses can effectively eliminate confounding variables and pinpoint causal factors (15).

Given the current uncertainty surrounding the causal relationships between micronutrients and pregnancy complications, our study utilized genome-wide association study (GWAS) data. The aim was to thoroughly investigate these potential causalities using a two-sample MR framework, supplemented by replication and meta-analyses to enhance the reliability of the MR estimates. Findings from this work would provide reliable evidence contributing to the development of strategies for pregnancy complications screening and preventions in clinical practice.

## 2 Materials and methods

This Mendelian randomization (MR) analysis utilized previously published GWAS summary statistics. All referenced data sources secured participant informed consent and acquired the necessary ethical approval. This study adhered to the Strengthening the Reporting of Observational Studies in Epidemiology Using Mendelian Randomization (STROBE-MR) reporting guideline (16).

### 2.1 Study design

**Figure 1:**
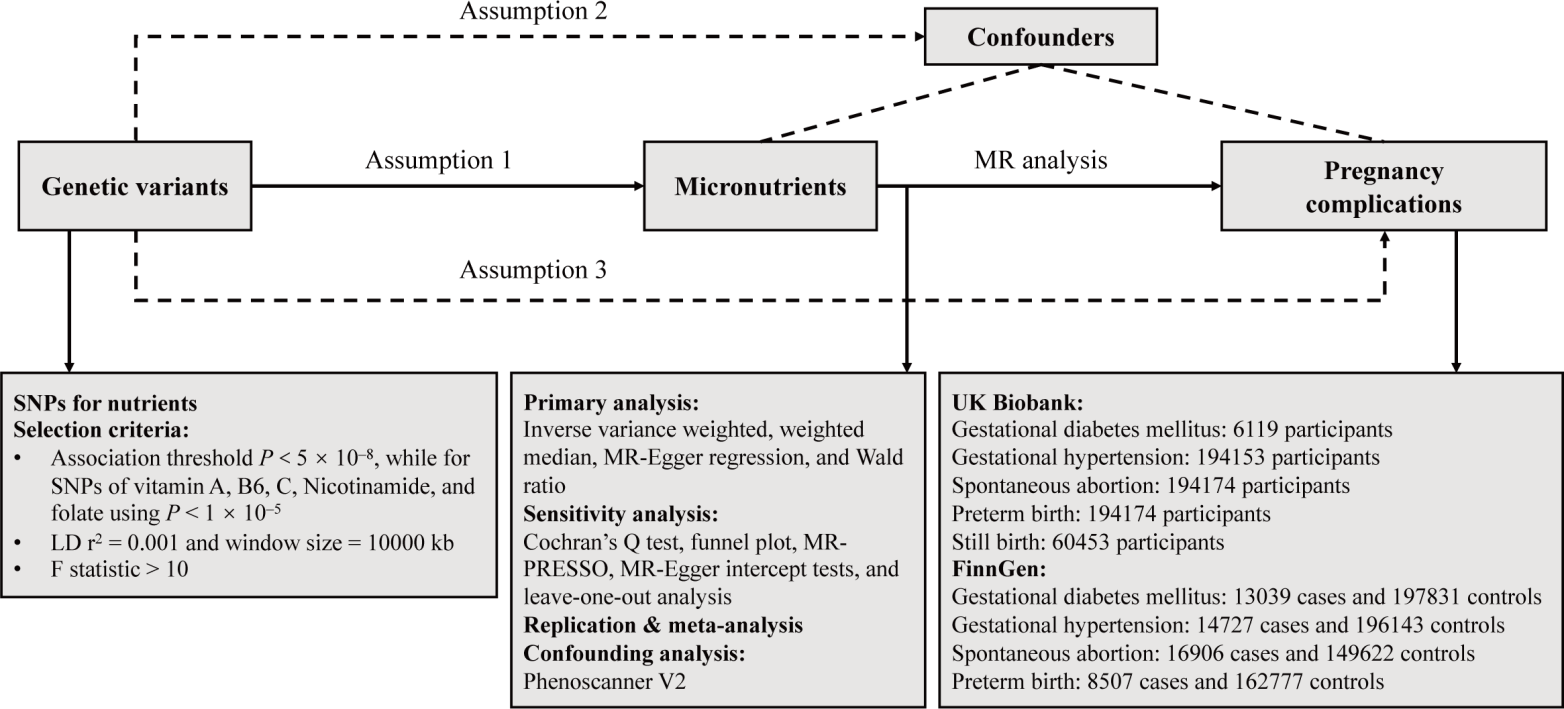
Overflow of the current Mendelian randomization (MR) study. SNPs, single nucleotide polymorphisms; LD, linkage disequilibrium.

We systematically examined the potential causal relationships between circulating minerals, vitamins, and the risk of pregnancy complications using a two-sample MR design. A robust MR design should meet three core assumptions: (1) genetic instruments exhibit strong associations with exposures; (2) genetic instruments have no associations with confounding variables; (3) genetic instruments influence outcomes only through the exposures of interest (17). The latter two assumptions are collectively termed the independence from horizontal pleiotropy and can be evaluated using various sensitivity analyses (18), such as Cochran’s Q statistic, MR-Egger intercept tests. Genetic data for pregnancy complications were sourced from the UK Biobank (UKB) and FinnGen consortia for primary and replication analyses. Subsequently, a meta-analysis was performed. Figure 1 provided an overview of the study. All statistical analyses were performed utilizing the TwoSampleMR package (Version 0.5.7) and MRPRESSO (Version 1.0) in R (Version 4.2.2), and the Reviewer Manager software (Version 5.3.3).

### 2.2 GWAS data for circulating minerals and vitamins

Given the prevalent use of Elevit as a nutrient supplement before and during pregnancy in Chinese clinics, we selected micronutrients based on Elevit’s composition to ascertain their direct causal effects on pregnancy complications. As the lack of GWAS data derived from Chinese or Asian ancestry, we undertook a comprehensive search of the most recent GWASs conducted on European populations, focusing on circulating minerals and vitamins. This search spanned the GWAS Catalog consortium and PubMed database. Subsequently, 14 exposures were identified: calcium (19), phosphorus (20), magnesium (19), iron (19, 21), zinc (21), copper (21), vitamin A (19), B6 (19), B12 (19), C (22), D (19), E (23), nicotinamide (23), and folate (19). Participants in the above GWAS studies were recruited mainly from United States, Netherlands, Australia, TwinsUK cohort, Germany, and Canada. This suggests that there may be no or merely minimal sample overlap with the outcome data of our study. Detailed information, such as recruitment criteria of population and quality control of genetic data, can be found in the original paper (Table 1). Unfortunately, no available GWASs were found for vitamins B1, B2, D3, biotin, and calcium pantothenate, hence they were excluded from this study. Although gender-stratified data was accessible from the UKB, it was not incorporated to prevent overlap of study samples.

**Table 1:** Details of the GWASs included in the Mendelian randomization.

	Traits	SNPs	Consortium	Participants case/control	Ancestry	PMID
Exposure	Calcium	8	GWAS Catalog	62,143	European	33441150
	Phosphorus	4		16,264		20558539
	Magnesium	2		20,707		33441150
	Iron	2		15,335		33441150
	Zinc	2		2,603		23720494
	Copper	2		2,603		23720494
	Vitamin A	12		2007		33441150
	Vitamin B6	14		1758		33441150
	Vitamin B12	5		19,415		33441150
	Vitamin C	13		7,824		24816252
	Vitamin D	2		18,315		33441150
	Vitamin E	1		8,192		36635386
	Nicotinamide	21		8,110		36635386
	Folate	11		5,998		33441150
Outcome	Gestational diabetes mellitus		UK Biobank	691/5428	European European	
			FinnGen	13,039/197831		
	Gestational hypertension		UK Biobank	1474/192679	European European	
			FinnGen	14,727/196143		
	Spontaneous abortion		UK Biobank	1150/193024	European European	
			FinnGen	16,906/149622		
	Preterm birth		UK Biobank	194,174	European European	
			FinnGen	8507/162777		
	Stillbirth		UK Biobank	60,453	European	

### 2.3 GWAS data for pregnancy complications

Five prevalent pregnancy complications: gestational diabetes mellitus (GDM), gestational hypertension (GH), spontaneous abortion (SA), preterm birth (PTB), and stillbirth (SB) were included as outcomes in this study (Table 1). Summary-level GWAS data from UK Biobank were used as discovery set in primary analysis, which were available at the website: http://www.nealelab.is/uk-biobank/. Additionally, GWAS data retrieved from the latest R9 release of the FinnGen consortium were used for replication (24). A GDM diagnosis was coded according to the ICD-9 (6480), ICD-10 (O24); a GH diagnosis was coded according to the ICD-8 (63701), ICD-9 (6423), ICD-10 (O13); a SA diagnosis was coded according to the ICD-8 (643), ICD-9 (634), ICD-10 (O03); a PTB diagnosis was coded according to the ICD-8 (63497), ICD-9 (644), ICD-10 (O60). However, the FinnGen consortium lacked GWAS data for SB.

### 2.4 Instruments selection

We implemented a meticulous process to select suitable genetic variants linked to circulating minerals and vitamins. Initially, due to a scarcity of SNPs for vitamin A, B6, C, nicotinamide, and folate that reached genome-wide significance, we adopted a relaxed association threshold of *p* < 1 × 10^−5^, which was in accordance with the study of Chen et al. (25). For other micronutrients, a more stringent *p* < 5 × 10^−8^ threshold was applied to identify the most independent SNPs. Clumping procedures were then applied using a linkage disequilibrium (LD) r^2^ of 0.001 and a window size of 10,000 kb. To mitigate bias from weak instruments, we calculated F statistics for each SNP, discarding those with an *F* < 10 to ensure a robust variance contribution for each nutrient (26, 27). Subsequently, we extracted the pertinent SNPs from the outcome datasets, excluding any with a direct association (*p* < 5 × 10^−8^). Harmonization followed, aligning exposure and outcome SNP alleles and removing palindromic SNPs with EAF > 0.42 or SNPs with mismatched alleles.

### 2.5 Primary analysis

For exposures with more than three SNPs, a random-effect inverse variance weighted (IVW) method was applied as the primary analysis, targeting significant causal effects with *p* < 0.05. Supplementary methods, including weighted median (WM) and MR-Egger regression, were used to corroborate the IVW findings. WM provides consistent estimates under the condition that over half of the information is free from horizontal pleiotropy (28), while MR-Egger regression can detect unbalanced pleiotropy and heterogeneity, though requiring a larger sample size (29). For exposures with two SNP instruments, a fixed-effects IVW approach was adopted, and for single-SNP exposures, the Wald ratio method was implemented.

### 2.6 Sensitivity analysis

To ascertain the robustness of our findings, we engaged several methods: Cochran’s Q statistic, MR-Egger intercept tests, Mendelian Randomization Pleiotropy Residual Sum and Outlier (MR-PRESSO), funnel plots, and leave-one-out (LOO) analysis. These were designed to uncover any heterogeneity, pleiotropy, outliers, and to validate the consistency of the results. MR-PRESSO and LOO analyses were restricted to cases with three or more instrumental variants.

### 2.7 Replication and meta-analysis

We further replicated our MR analysis using independent GWAS data from the FinnGen consortium and performed meta-analysis to confirm the final results. However, due to the absence of GWAS data for SB in FinnGen, we could not perform replication and meta-analysis for the causal association between micronutrients and SB.

### 2.8 Confounding analysis

In addition to the comprehensive statistical methods employed in the sensitivity analysis to scrutinize potential MR assumption violations, we explored the association of exposure-related SNPs with common confounding risk factors via the Phenoscanner V2 platform.^1^ SNPs associated with confounders at *p* < 1 × 10^−5^ were excluded to prevent any bias in the MR estimates.

## 3 Results

### 3.1 Screening of instruments

Following the instrument selection criteria, 8 SNPs of calcium, 4 SNPs of phosphorus, 2 SNPs of magnesium, iron, zinc, copper, and vitamin D, 12 SNPs of vitamin A, 14 SNPs of vitamin B6, 5 SNPs of vitamin B12, 13 SNPs of vitamin C, 1 SNP of vitamin E, 21 SNPs of nicotinamide, and 11 SNPs of folate in circulating micronutrients were included in MR analysis. F statistics for SNPs after clumping were consistently above 10, indicating the absence of weak instruments in our study (Supplementary Table S1). Harmonized data for each outcome in discovery set and replication set are detailed in Supplementary Tables S2, S3.

### 3.2 MR estimates

**Figure 2:**
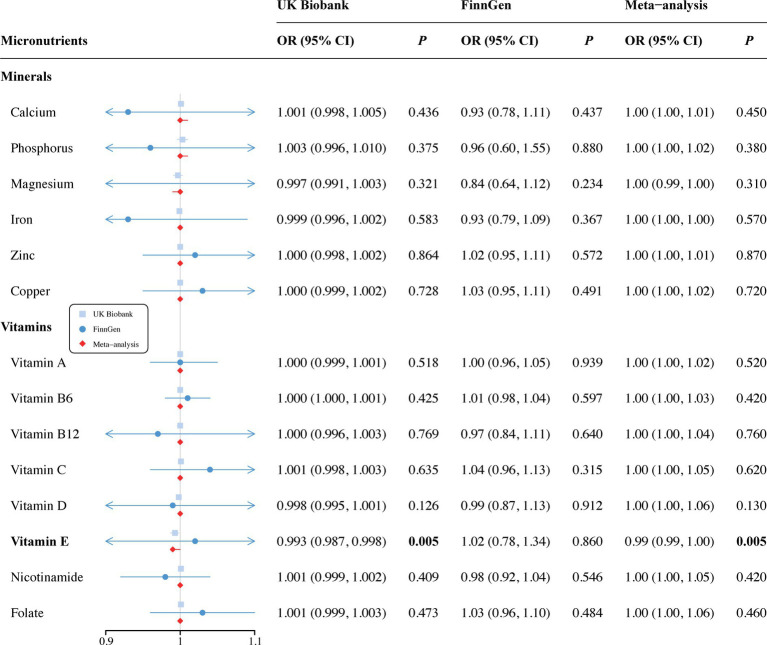
Associations of minerals and vitamins with risk of spontaneous abortion. CI, confidence interval; OR, odds ratio.

**Figure 3:**
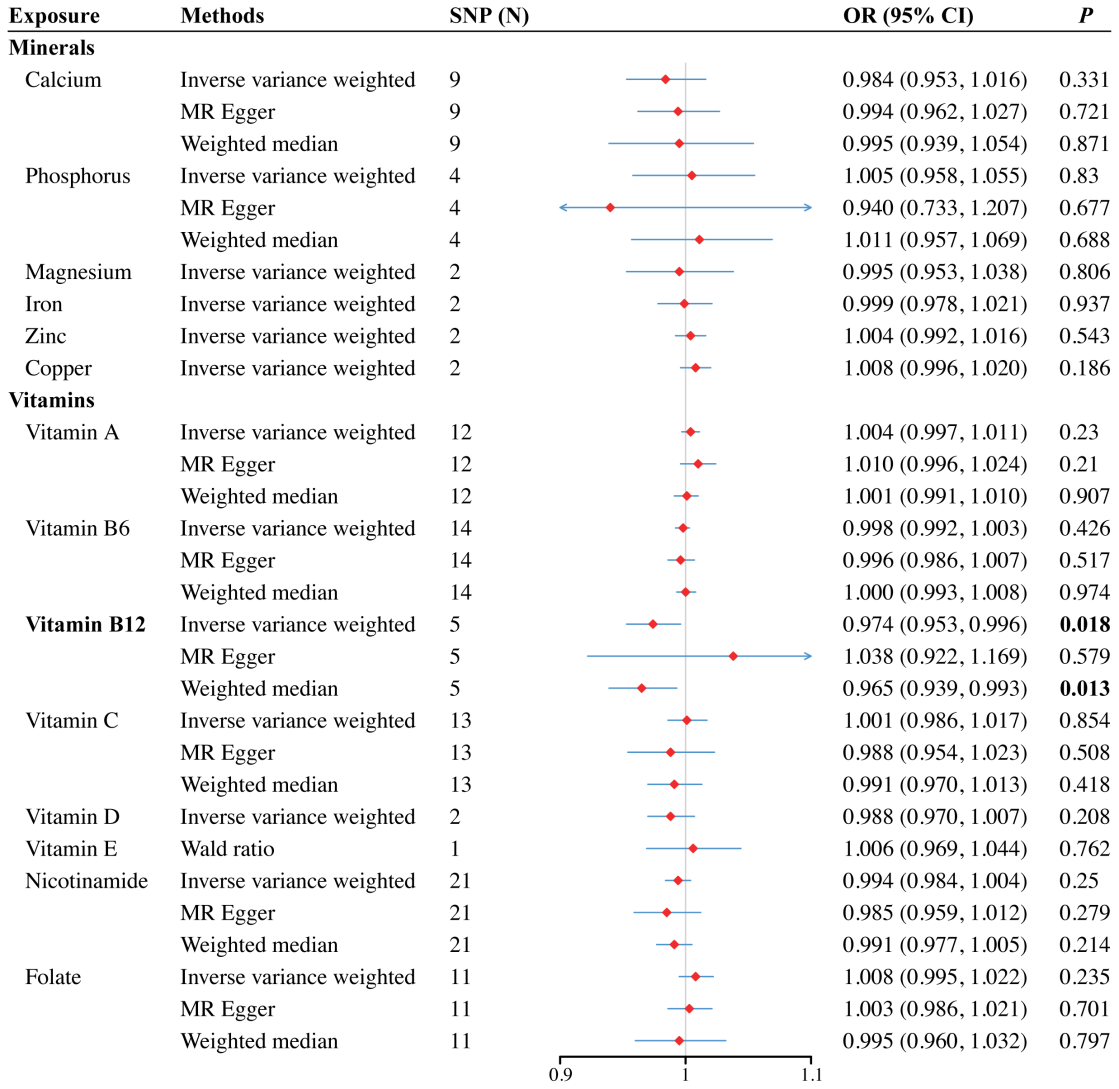
Forest plot for the causal effects of minerals and vitamins on the risk of still birth derived from inverse variance weighted (IVW), weighted median (WM) and MR-Egger analysis. CI, confidence interval; OR, odds ratio.

In the vitamin E phenotype, Wald ratio analysis showed that genetically predicted elevated circulating vitamin E levels correlated with a decreased risk of SA in the discovery set (OR = 0.993, 95% CI 0.987–0.998; *p* = 0.005) (Figure 2). Additionally, potential causality was observed between genetic predisposition for vitamin B12 and SB, as identified by both IVW (OR = 0.974, 95% CI 0.953–0.996; *p* = 0.018) and WM analyses (OR = 0.965, 95% CI 0.939–0.993; *p* = 0.013) (Figure 3). However, no causal relationships were observed between other analyzed circulating minerals or vitamins and pregnancy complications (Supplementary Tables S4, S5). Especially, no associations were identified between circulating folate levels and risks of SA (OR = 1.001, 95% CI 0.999–1.003; *p* = 0.473), PTB (OR = 1, 95% CI 1–1.001; *p* = 0.297), or SB (OR = 1.008, 95% CI 0.995–1.022; *p* = 0.235). Besides, none of the analyzed minerals or vitamins were found to play a causal role in the onset of GDM, GH, or PTB.

### 3.3 Sensitivity analyses

To validate our primary findings, we conducted several sensitivity analyses, including the Cochran’s Q test, MR-Egger intercept, and the MR-PRESSO global test. Notably, we could not conduct sensitivity analyses for genetically inferred vitamin E levels as only one SNP were included in MR analysis. No significant heterogeneity, pleiotropy, or outliers were identified in the association between vitamin B12 and SB (Q value = 3.007, *p* = 0.56; Intercept = −0.005, *p* = 0.36; MR-PRESSO *p* = 0.593). Besides, the LOO analysis further confirmed that no individual SNP heavily influenced the result (Supplementary Figure S5). The symmetry observed in the funnel plot (Supplementary Figure S8) also supported the integrity of our estimate. Comprehensive results from the sensitivity analyses can be found in Supplementary Tables S4, S5, with the funnel plots, scatter plots, and LOO results illustrated in Supplementary Figures S1–S16.

### 3.4 Replication and meta-analysis

Since only 1 SNP for vitamin E was included in the MR analysis, we used the independent GWAS data from the FinnGen consortium for validation and performed a meta-analysis to further confirm our findings. We did not observe a causal relationship between vitamin E and SA in replication set (OR = 1.02, 95% CI 0.78–1.34; *p* = 0.860). However, a combined analysis of both datasets supported the causality (OR = 0.99, 95% CI 0.99–1.00; *p* = 0.005) (Figure 2). Unfortunately, publicly available GWAS data on SB were not found during the search and therefore, the causal association between vitamin B12 and SB could not be validated. Additionally, no new causalities between other vitamins, minerals and pregnancy complications were found during the replication and meta-analysis. Details are listed in Supplementary Table S5.

### 3.5 Confounding analysis

Despite the rigorous sensitivity analyses, we further examined the secondary traits of the harmonized SNPs using the Phenoscanner. This review revealed that although SNPs related to vitamins E and B12 were not linked with any confounders, the SNP rs1728918 for calcium showed a connection with diabetes. Similarly, for iron, the SNP rs1800562 was associated with factors like blood pressure medication, self-reported hypertension, and cardiovascular disease. Importantly, our primary estimates remained consistent after excluding these specific SNPs (Supplementary Tables S4, S5).

## 4 Discussion

**Figure 4:**
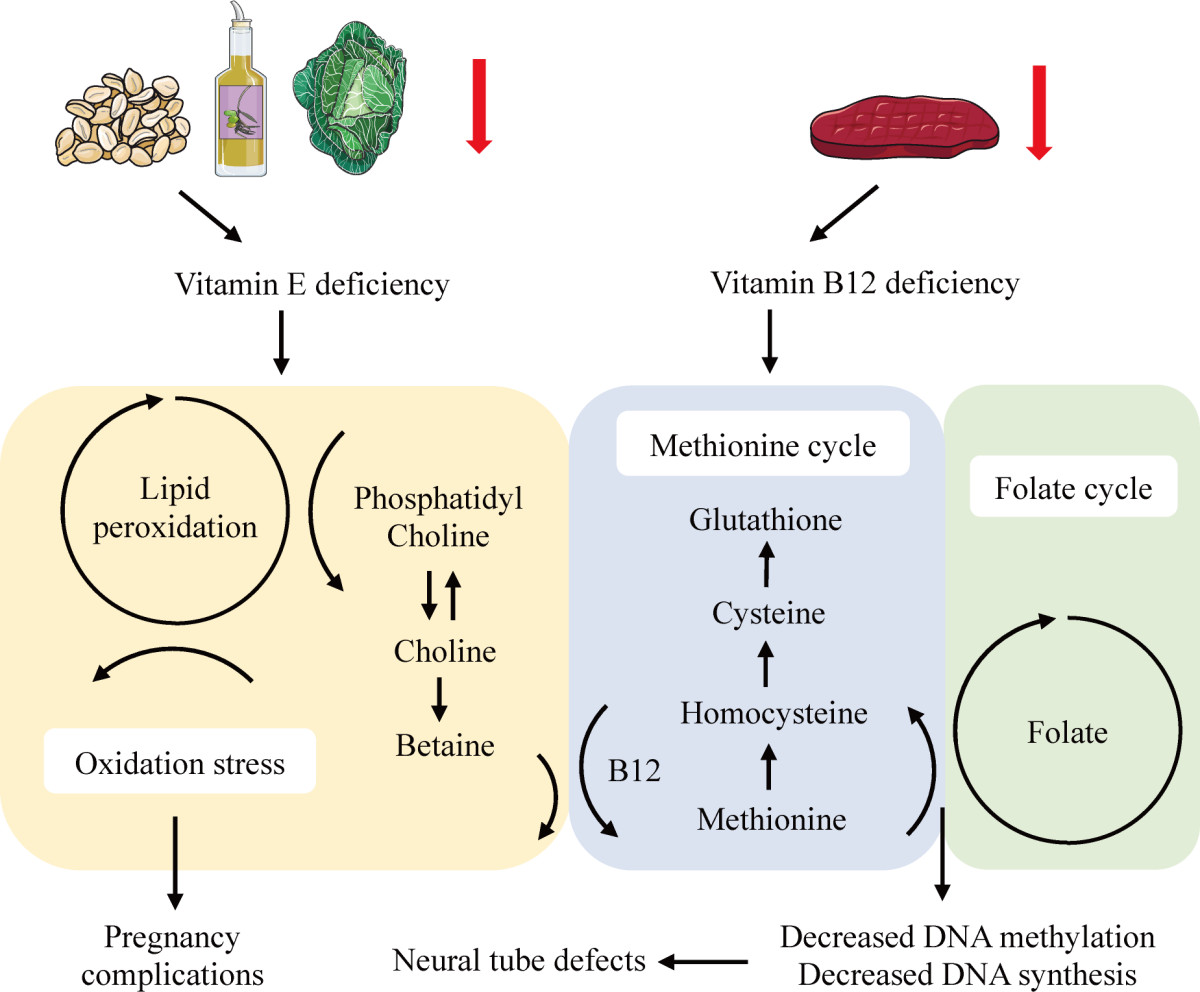
Systemic effects of vitamin E and B12 deficiency in the lipid peroxidation and one-carbon metabolism.

In our study, we established a definitive genetic link between elevated levels of circulating vitamin E and a reduced risk of spontaneous abortion (SA). Additionally, an increased genetic predisposition for vitamin B12 was suggested to confer a protective effect against stillbirth (SB). We observed no associations between folate and risks for SA, preterm birth (PTB), or SB. Likewise, no minerals or vitamins demonstrated a causal role in the onset of gestational diabetes mellitus (GDM), gestational hypertension (GH), or PTB. To our knowledge, this is the first Mendelian randomization (MR) study to use replication and meta-analysis for a comprehensive evaluation of the causative role of micronutrients in pregnancy complications (see Figure 4).

The significant impact of pregnancy complications on both immediate and long-term maternal and neonatal health underscore the critical need for early screening and preventive measures. Although various studies have implicated micronutrients in adverse pregnancy outcomes, the inconsistencies stemming from demographic and methodological differences present a challenge for clinical guidance. For instance, a Chinese birth cohort study suggested that the effects of folate and multivitamin supplementation on birth defects vary by subtype, advocating for moderation in their use to mitigate the risk of birth defects (30). Inspired by the MR analysis conducted by Rogne et al. (31), our multi-exposure MR study aims to provide robust evidence for the implementation of nutritional supplementation and complication screening strategies for women of reproductive age.

Consistent with existing literature, our findings suggested an inverse correlation between circulating vitamin E levels and the risk of SA (32, 33). Specifically, a large-scale cohort study in rural Bangladesh linked lower vitamin E levels in early pregnancy to a heightened risk of miscarriage (32), while a single-blind RCT indicated that vitamin E supplementation could enhance uterine artery blood flow, potentially benefiting women with a history of recurrent abortion due to circulatory impairments (33). While the precise pathophysiological mechanisms underpinning the relationship between vitamin E and SA are not fully understood, several hypotheses have emerged. For instance, experiments on zebrafish models have demonstrated that vitamin E deficiency leads to increased lipid peroxidation in phosphatidylcholine-docosahexaenoic acid (DHA-PC), resulting in choline depletion and increased betaine production, which disrupts the methionine cycle (34). This disruption may also affect the one-carbon metabolism, involving folate and vitamin B12, potentially precipitating neural tube defects and other pregnancy-related complications via diminished DNA methylation and synthesis (35, 36). Furthermore, vitamin E is known to protect polyunsaturated fatty acids (PUFAs) and their lipid mediators from lipid peroxidation, and inhibit lipoxygenases (LOX), thus reducing apoptosis (37). Conversely, a recent meta-analysis casts doubt on the benefits of routine vitamin E supplementation during pregnancy, hinting at possible adverse effects (38). These findings, which suggest variable impacts of vitamin E based on its concentration and the individual’s nutritional status (39–41), highlight the need for further clinical trials with consistent methodologies and well-defined vitamin status classifications (42).

In terms of vitamin B12, our MR findings pointed to a tentative causal link with SB risk, consistent across both the IVW and WM methods. Although replication and meta-analysis were constrained by the absence of SB data in the FinnGen consortium, the evidence, when viewed in conjunction with prior studies (43, 44), suggested a potential causative association between vitamin B12 and SB. Further well-designed RCTs and larger GWAS datasets will be beneficial for validation of the result.

However, we did not identify associations with folate nor with any other minerals or vitamins for the selected pregnancy complications. This contrasts with some observational studies that have suggested such links (11, 45–47), potentially attributable to the inherent confounders and biases of observational design.

Literatures on supplementation of micronutrients are limited to a few, small-scale studies that only target specific subgroups of patients, precluding specific recommendations. Findings from this study identified a causal association between circulating vitamins and pregnancy complications, enriching the research on nutritional intervention strategies for maternal and neonatal health.

The main strength of this study is the MR design, which incorporated data from large consortia to provide solid genetic evidence for the reported associations. Specifically, an array of methods was performed in MR and sensitivity analysis to confirm the validity of our findings and adherence to MR assumptions. Second, by utilizing data from populations of European ancestry, we reduced confounding effects, reverse causality, and bias from population stratification. In addition, meta-analysis of two independent databases, covering four pregnancy complications, lends credence to the consistency and non-random nature of our results.

Limitations of this MR investigation also merit consideration. First, a stringent *p*-value cutoff (5e - 08) for most exposures was applied to diminish weak instrument bias, which might lead to an underpowered analysis susceptible to false negatives. Second, the genetic instruments for calcium and phosphorus explained a limited amount of variance in their blood levels, suggesting that negative findings should be interpreted with caution, as causality may not be fully excluded. Third, although we used female-specific outcome GWAS data, exposures were derived from combined-gender GWAS due to the lack of gender-stratified data. Moreover, small sample sizes for GWAS on certain micronutrients, namely zinc, copper, vitamin A, and B6, impeded the capacity to provide precise and clinically applicable conclusions. Future research would benefit from gender-specific and larger MR analyses to bolster the accuracy of the findings. Another limitation is that other antioxidants such as selenium and lycopene were not included in our study, which may be associated with certain health outcomes (48–50). A further limitation is that micronutrients generally showed a U-shaped correlation with various disease risks, emphasizing the need for caution in interpreting our results. Future research could explore alternative methods to validate our findings. Finally, we did not apply a correction for multiple testing. Instead, we chose for replication and meta-analysis to confirm our results, avoiding the risk of obscuring significant associations due to an overly stringent approach to multiple testing.

## 5 Conclusion

In summary, this MR analysis provided robust evidence that higher levels of circulating vitamin E might play a protective role in the progression of SA, while an increased genetic propensity for vitamin B12 could potentially decrease the likelihood of SB. Contrarily, we did not identify an association between folate levels and the risks for SA, PTB, or SB, nor did we find any causal relationships between other assessed micronutrients and GDM, GH, or PTB. These findings underscore the causal associations between circulating micronutrients and pregnancy complications, guiding clinicians in micronutrient supplementation decisions for women of childbearing age, especially those at high risk of nutritional deficiencies.

## Data availability statement

The datasets presented in this study can be found in online repositories. The names of the repository/repositories and accession number(s) can be found in the article/Supplementary material.

## Ethics statement

The studies involving humans were approved by the ethics committee which can be found in the original paper. The studies were conducted in accordance with the local legislation and institutional requirements. Written informed consent for participation was not required from the participants or the participants' legal guardians/next of kin in accordance with the national legislation and institutional requirements.

## Author contributions

YX: Conceptualization, Data curation, Formal analysis, Visualization, Project administration, Writing – original draft, Writing – review & editing. JZ: Funding acquisition, Writing – review & editing. SN: Writing – review & editing. JL: Funding acquisition, Writing – review & editing.

## Footnotes

^1^http://www.phenoscanner.medschl.cam.ac.uk/
