# Supplementary material for: Association of circulating minerals and vitamins with pregnancy complications: a Mendelian randomization study
Source: Front Nutr. 2024 Jun 18;11:1334974. doi: 10.3389/fnut.2024.1334974 (PMC11217313; doi:10.3389/fnut.2024.1334974)
Supplement: Supplementary file 2 [file Data_Sheet_2.ZIP › Figrure S9-16.docx]

Supplementary Material


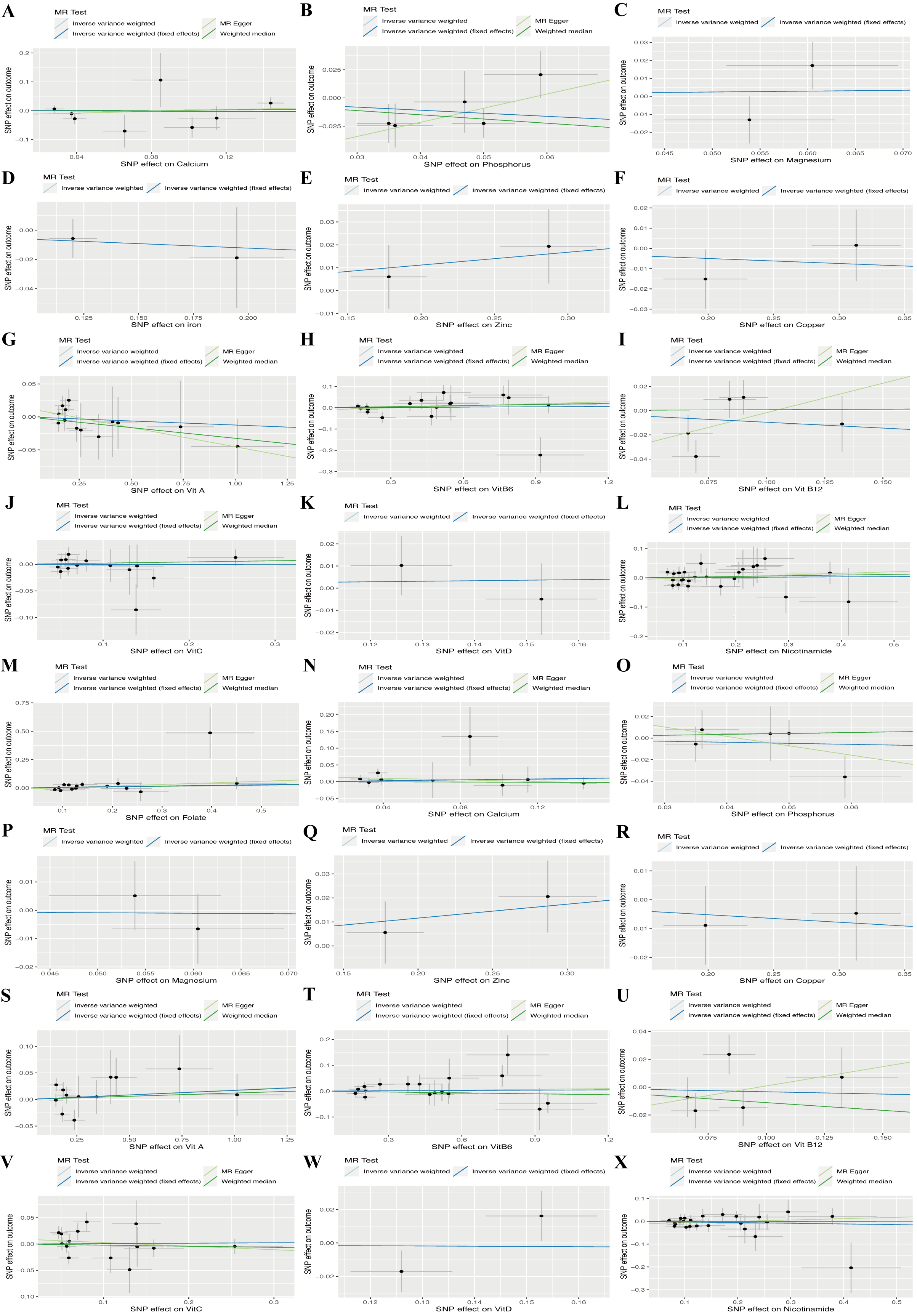


Figure S9. Scatter plots of the replication set. (A-M) Exposures (Ca, P, Mg, Fe, Zn, Cu, vit A, B6, B12, C, D, nicotinamide, folate) and gestational diabetes mellitus; (N-X) Exposures (Ca, P, Mg, Zn, Cu, vit A, B6, B12, C, D, nicotinamide) and gestational hypertension.


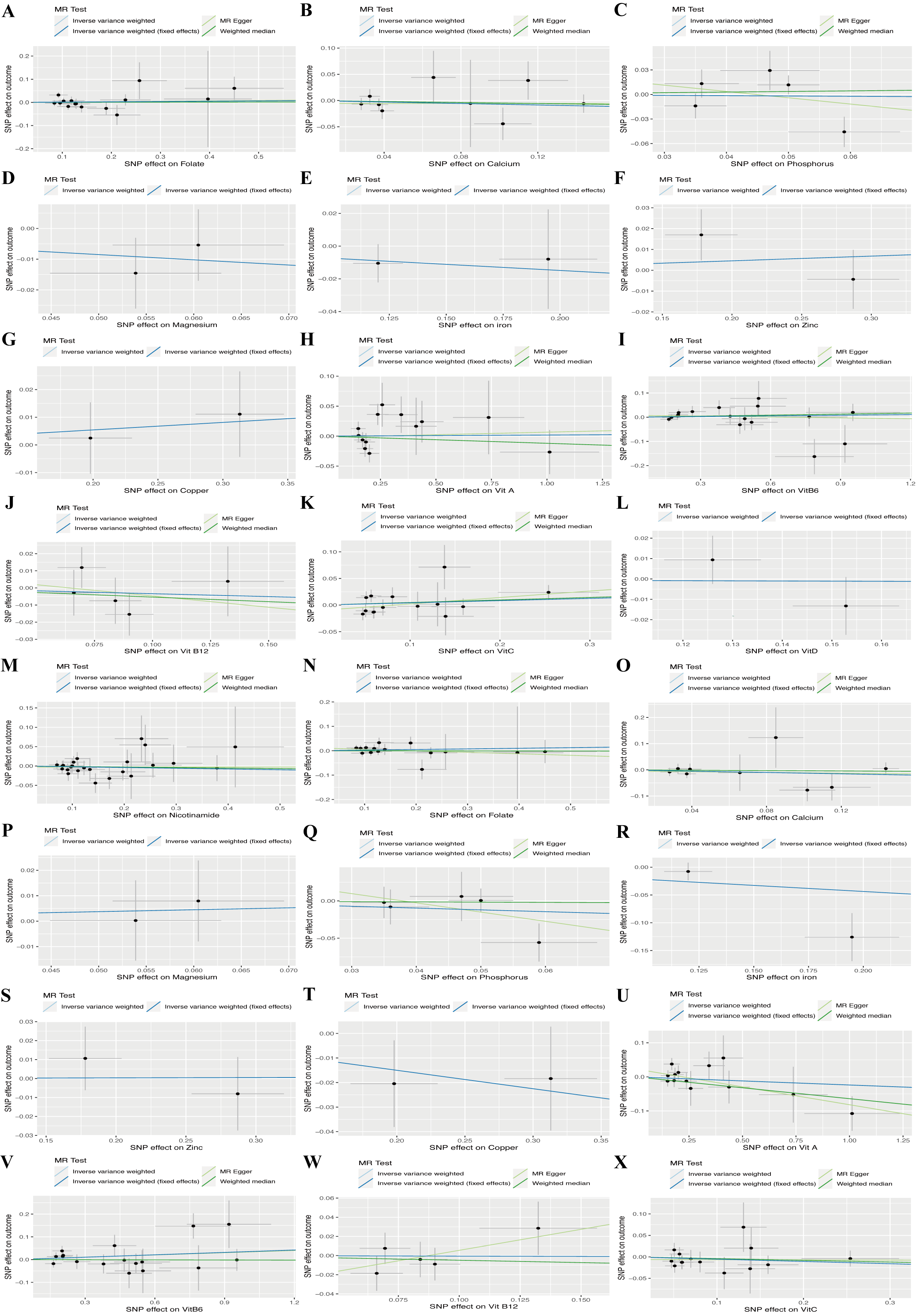


**Figure S10.** Scatter plots of the replication set. (A) Exposures (folate) and gestational hypertension; (B-N) Exposure (Ca, P, Mg, Fe, Zn, Cu, vit A, B6, B12, C, D, nicotinamide, folate) and spontaneous abortion; (O-X) Exposure (Ca, Mg, P, Fe, Zn, Cu, vit A, B6, B12, C) and preterm birth.


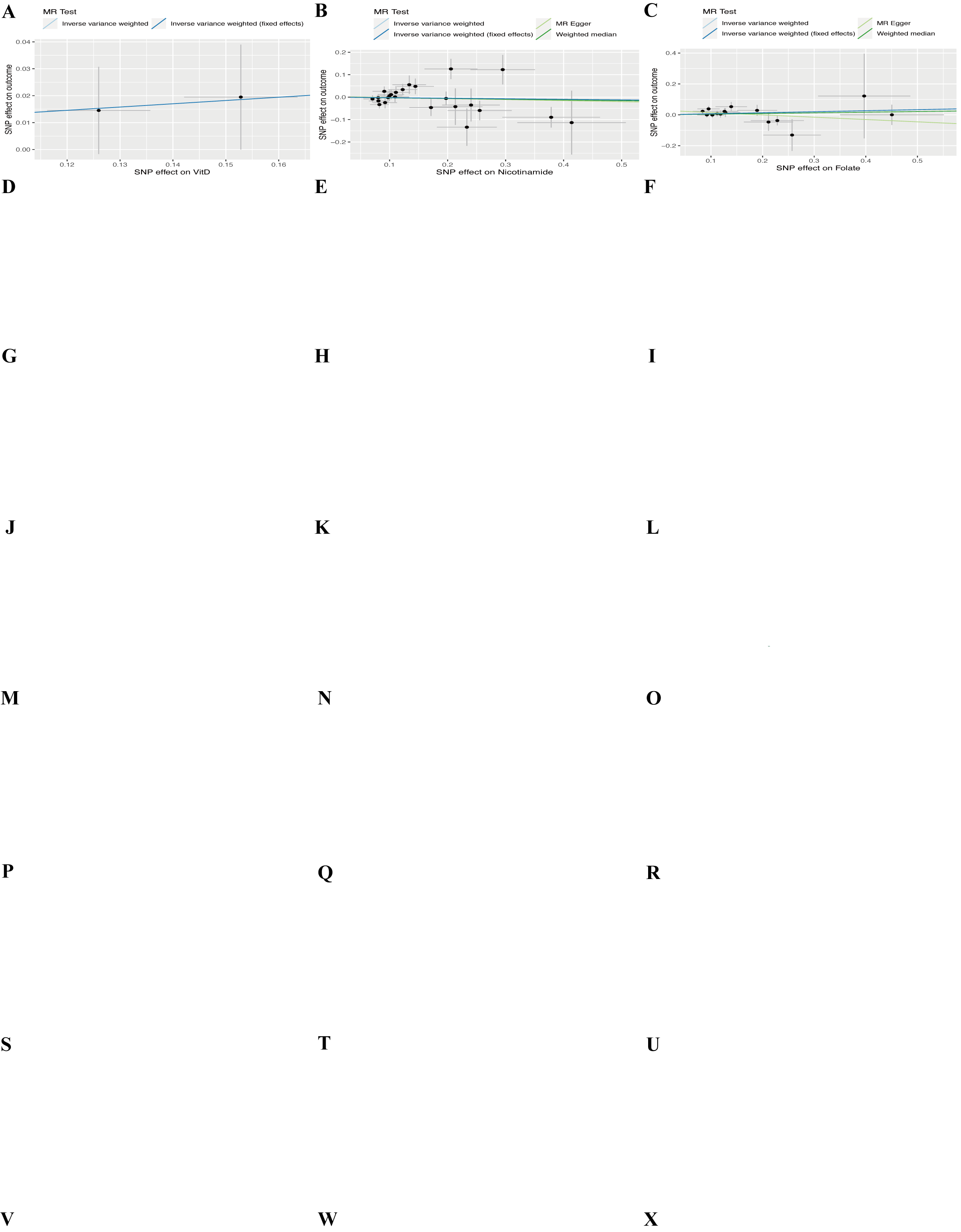


**Figure S11.** Scatter plots of the replication set. (A-C) Exposures (Vit D, nicotinamide, folate) and preterm birth.


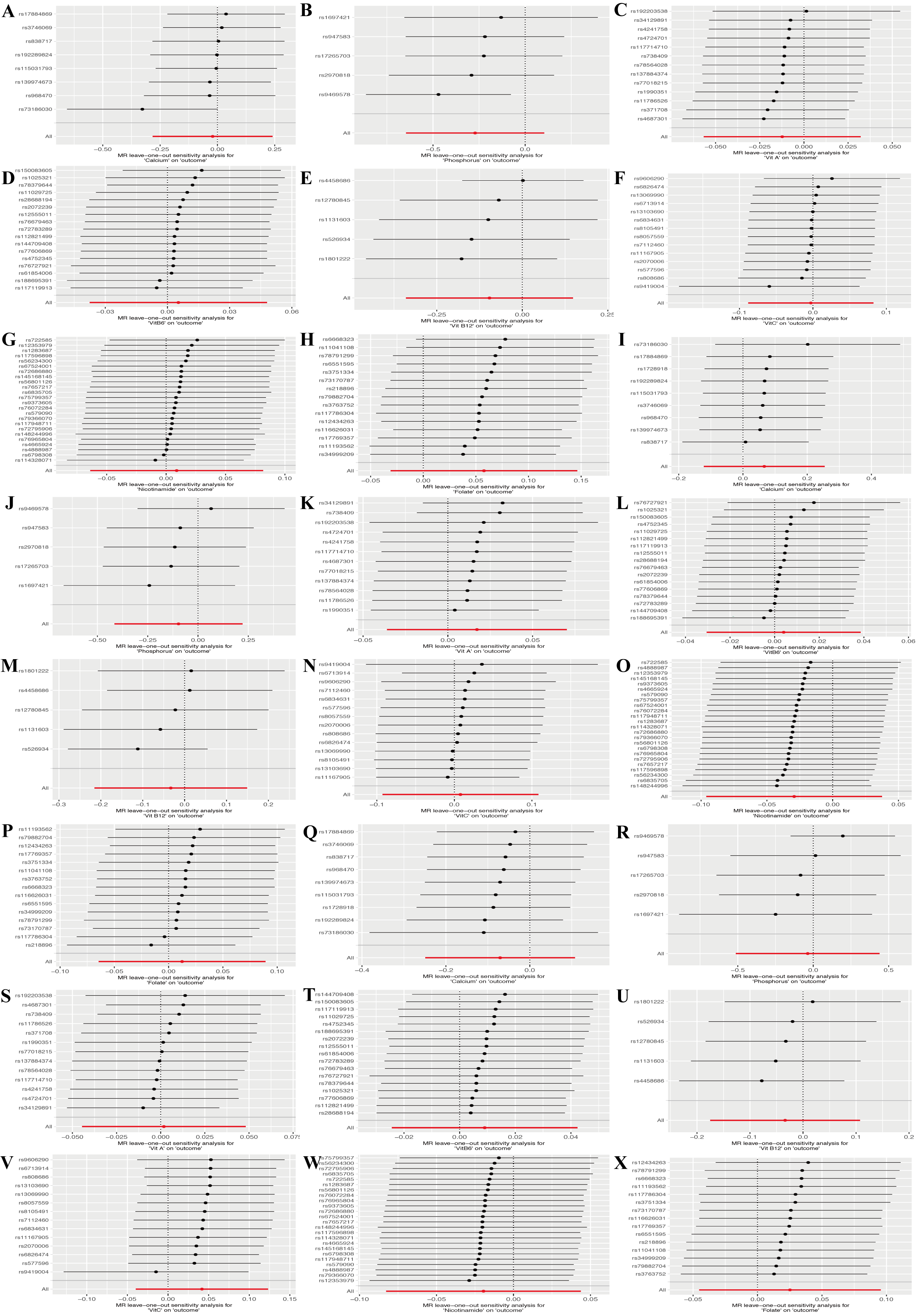


**Figure S12.** Leave-one-out sensitivity analysis of the replication set. (A-H) Exposures (Ca, P, vit A, B6, B12, C, nicotinamide, folate) and gestational diabetes mellitus; (I-P) Exposures and gestational hypertension; (Q-X) Exposures and spontaneous abortion.


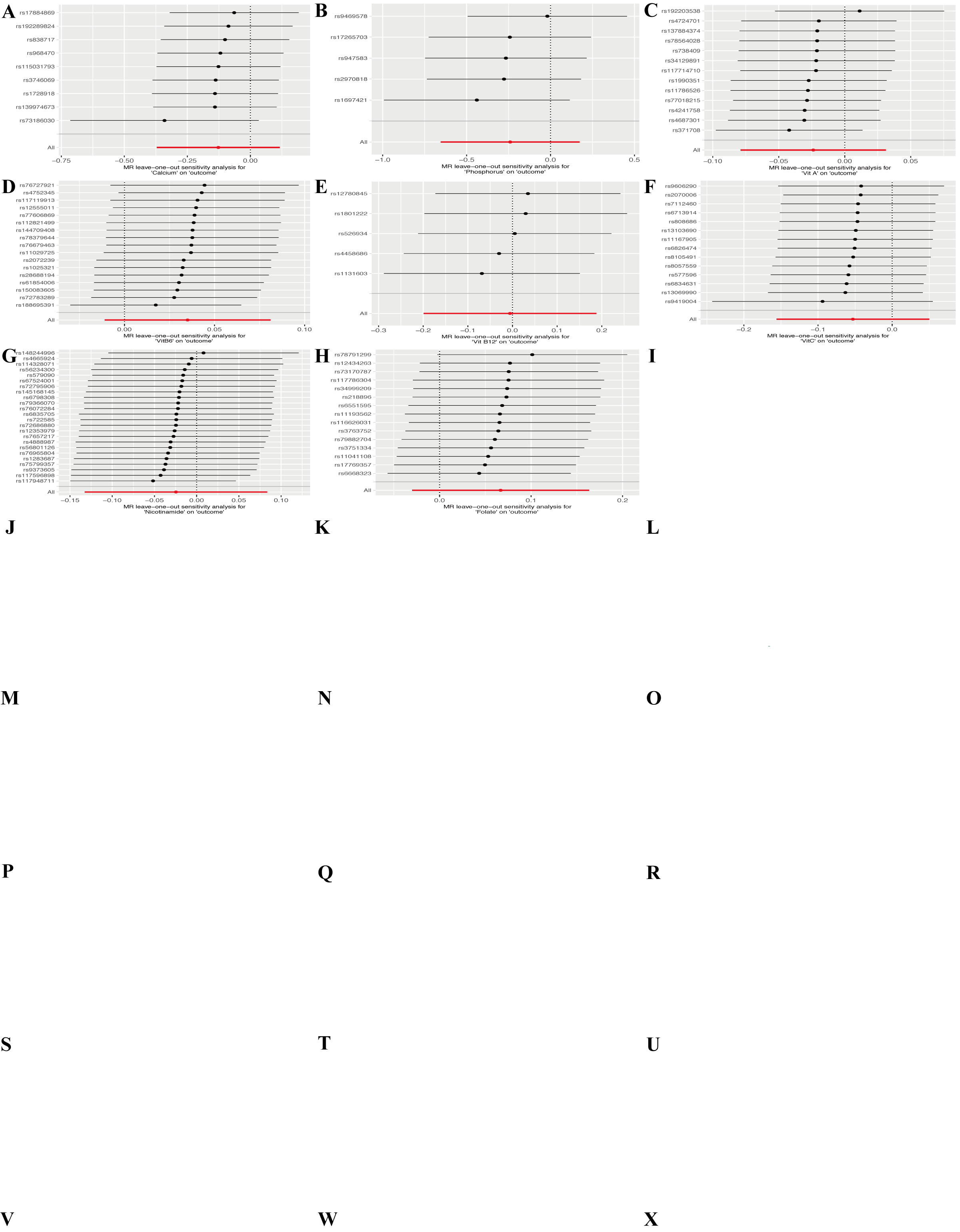


**Figure S13.** Leave-one-out sensitivity analysis of the replication set. (A-H) Exposures (Ca, P, vit A, B6, B12, C, nicotinamide, folate) and preterm birth.


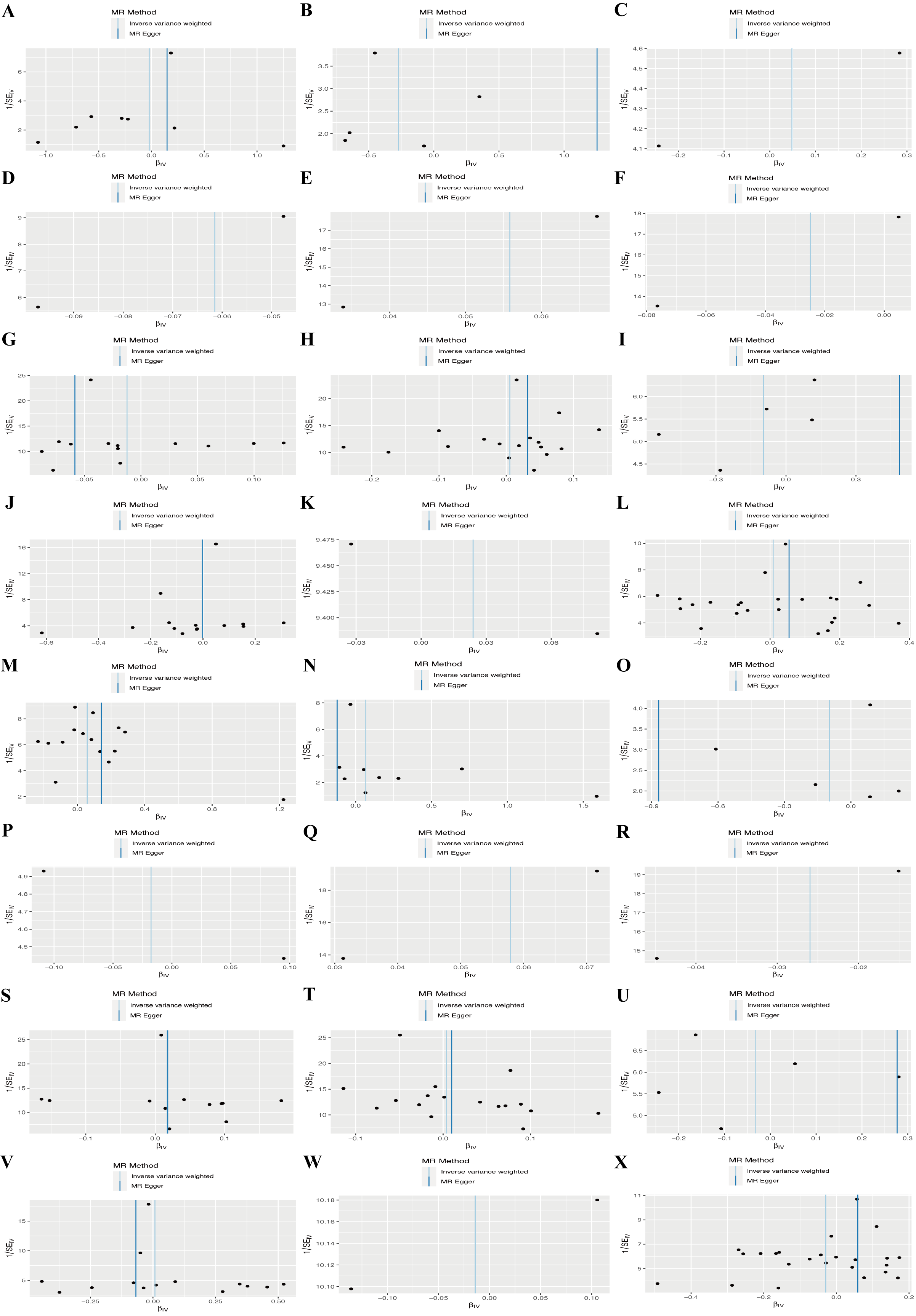


**Figure S14.** Funnel plots of the replication set. (A-M) Exposures (Ca, P, Mg, Fe, Zn, Cu, vit A, B6, B12, C, D, nicotinamide, folate) and gestational diabetes mellitus; (N-X) Exposures (Ca, P, Mg, Zn, Cu, vit A, B6, B12, C, D, nicotinamide) and gestational hypertension.


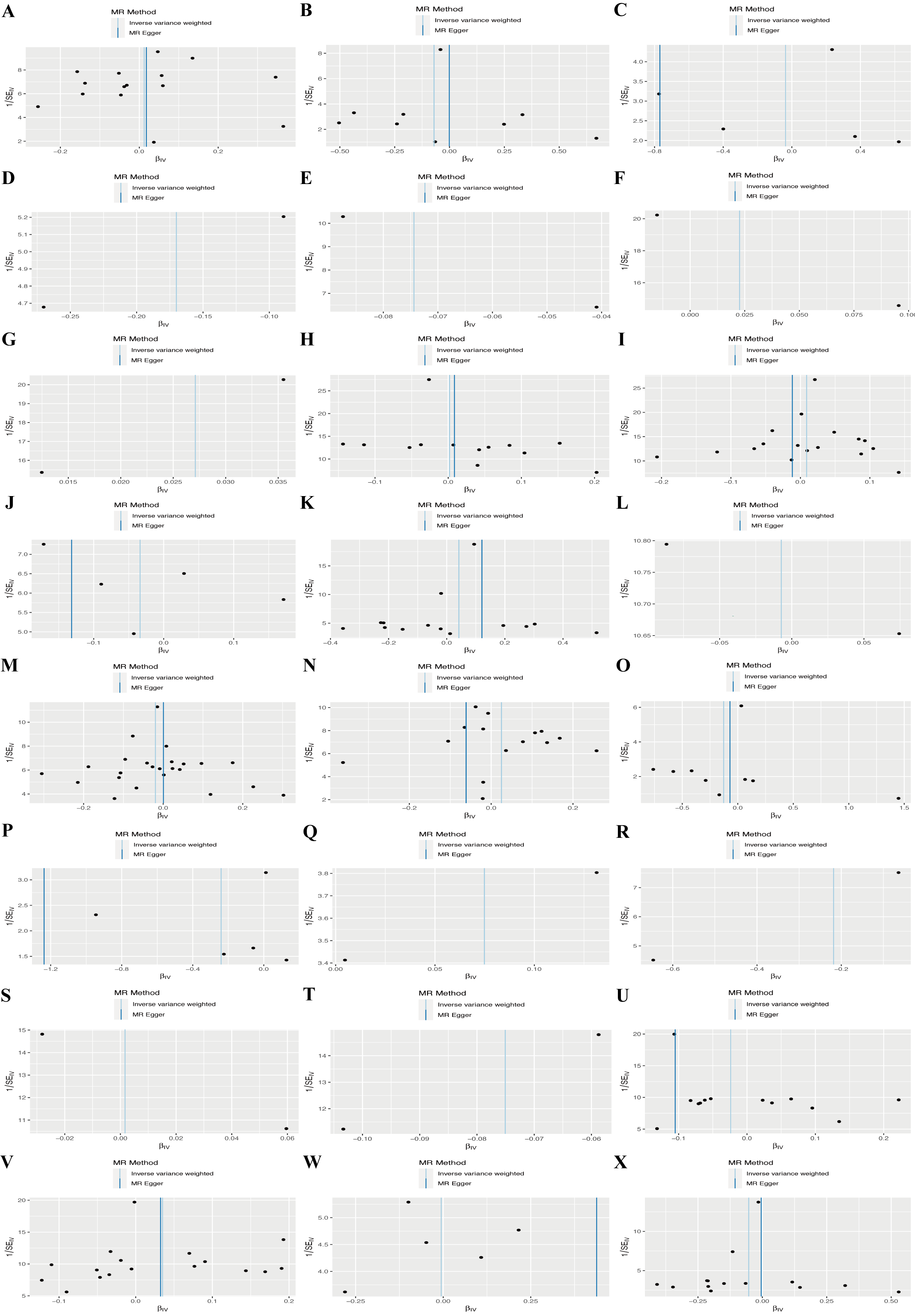


**Figure S15.** Funnel plots of the replication set. (A) Exposures (Folate) and gestational hypertension; (B-N) Exposures (Ca, P, Mg, Fe, Zn, Cu, vit A, B6, B12, C, D, nicotinamide, folate) and spontaneous abortion; (O-X) Exposures (Ca, P, Mg, Fe, Zn, Cu, vit A, B6, B12, C) and preterm birth.


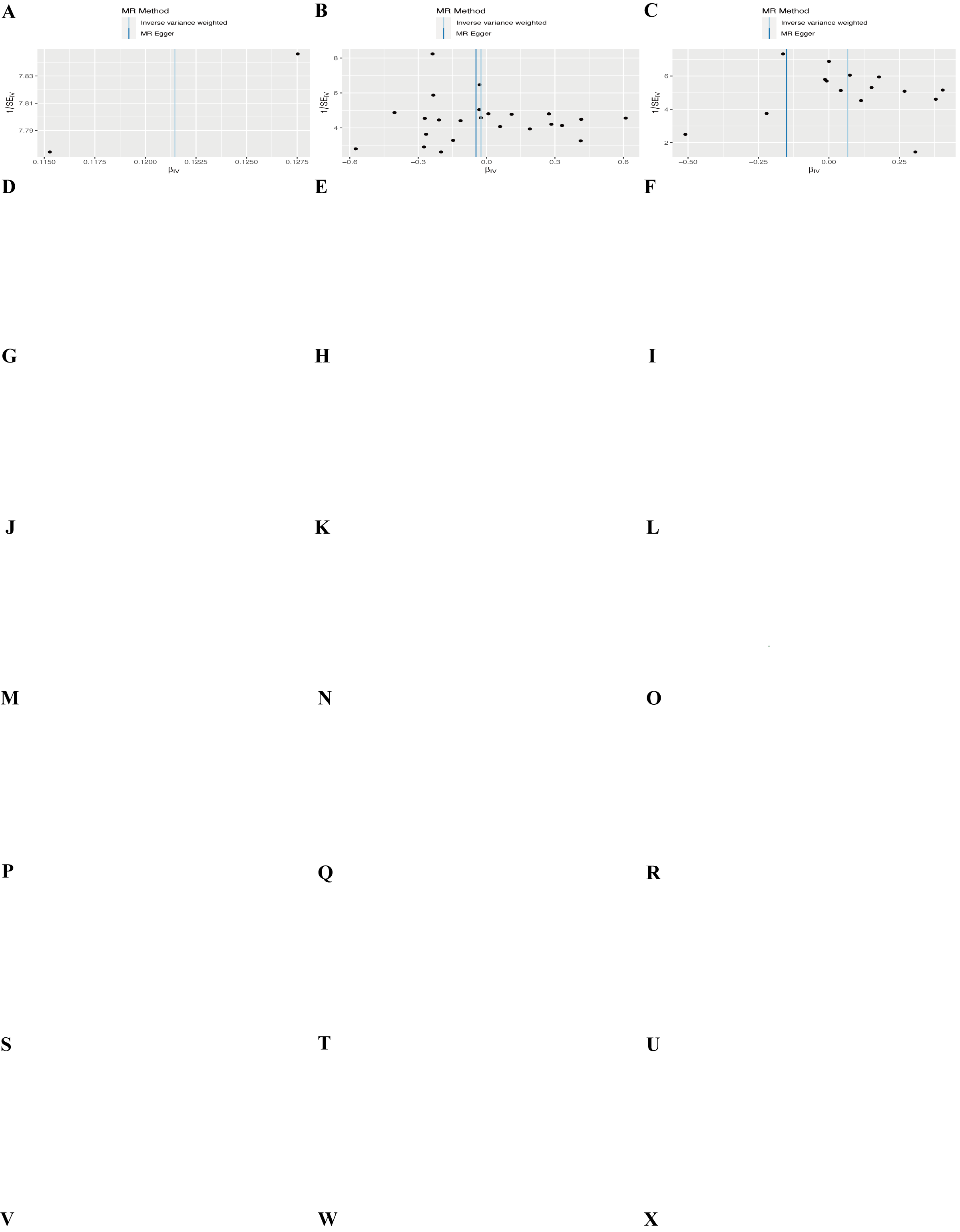


**Figure S16.** Funnel plots of the replication set. (A-C) Exposures (Vit D, nicotinamide, folate) and preterm birth.
