# Supplementary material for: Association of circulating minerals and vitamins with pregnancy complications: a Mendelian randomization study
Source: Front Nutr. 2024 Jun 18;11:1334974. doi: 10.3389/fnut.2024.1334974 (PMC11217313; doi:10.3389/fnut.2024.1334974)
Supplement: Supplementary file 2 [file Data_Sheet_2.ZIP › Figrure S1-8.docx]

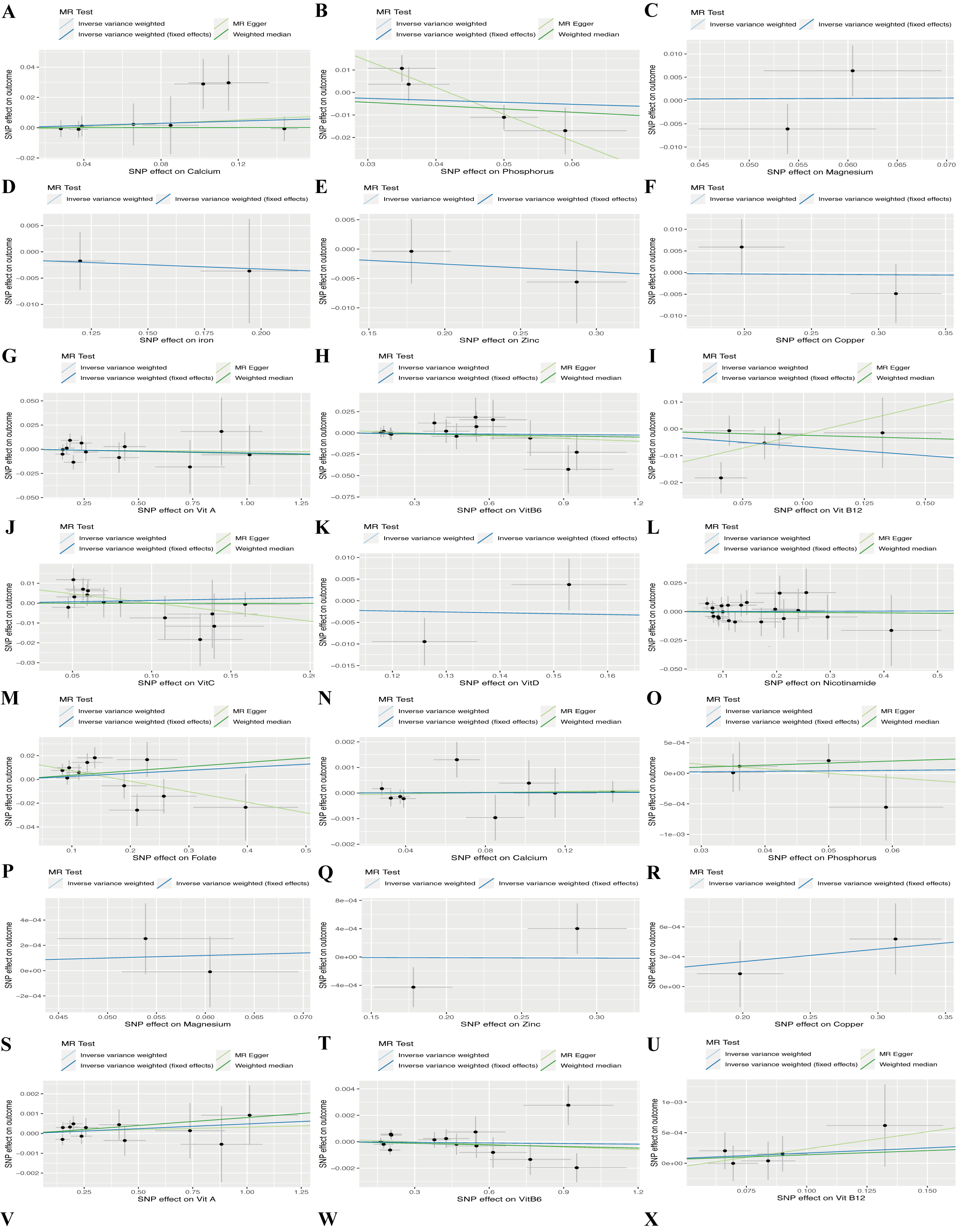
Supplementary Material

Figure S1. Scatter plots of the discovery set. (A-M) Exposures (Ca, P, Mg, Fe, Zn, Cu, vit A, B6, B12, C, D, nicotinamide, folate) and gestational diabetes mellitus; (N-U) Exposures (Ca, P, Mg, Zn, Cu, vit A, B6, B12) and gestational hypertension.


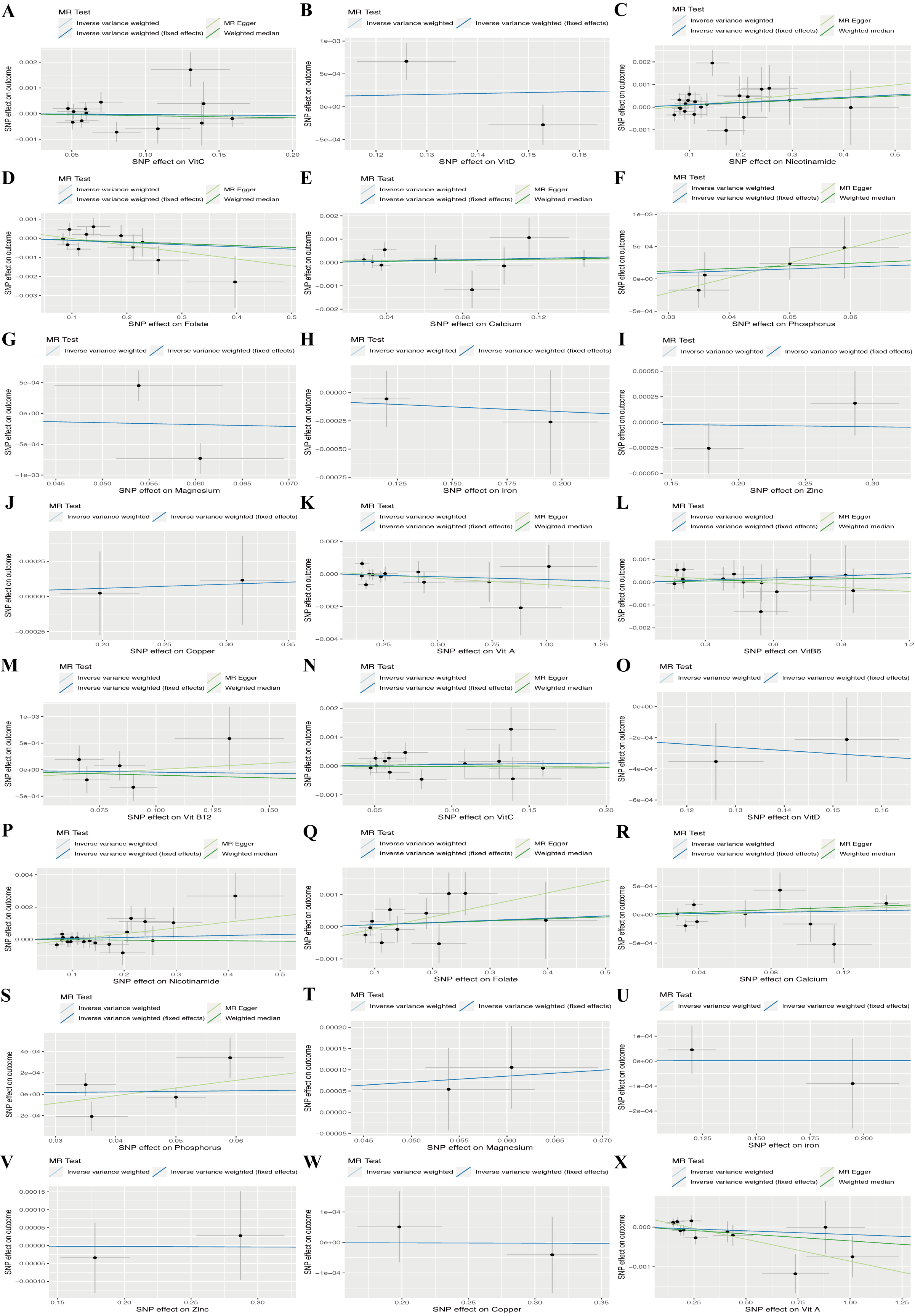


**Figure S2.** Scatter plots of the discovery set. (A-D) Exposures (Vit C, D, nicotinamide, folate) and gestational hypertension; (E-Q) Exposure (Ca, P, Mg, Fe, Zn, Cu, vit A, B6, B12, C, D, nicotinamide, folate) and spontaneous abortion; (R-X) Exposure (Ca, P, Mg, Fe, Zn, Cu, vit A) and preterm birth.


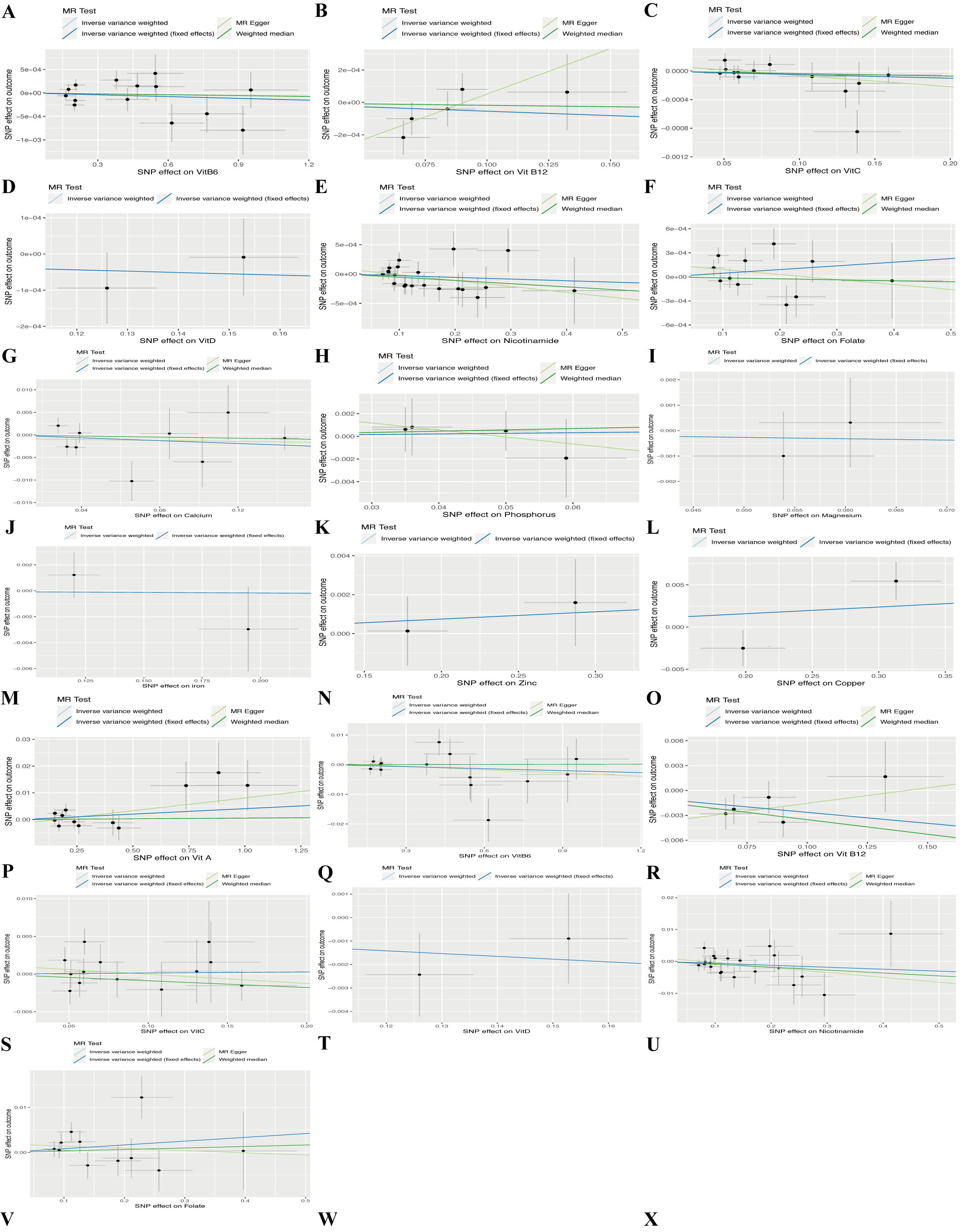


**Figure S3.** Scatter plots of the discovery set. (A-F) Exposures (Vit B6, B12, C, D, nicotinamide, folate) and preterm birth; (G-S) Exposure (Ca, P, Mg, Fe, Zn, Cu, vit A, B6, B12, C, D, nicotinamide, folate) and still birth.


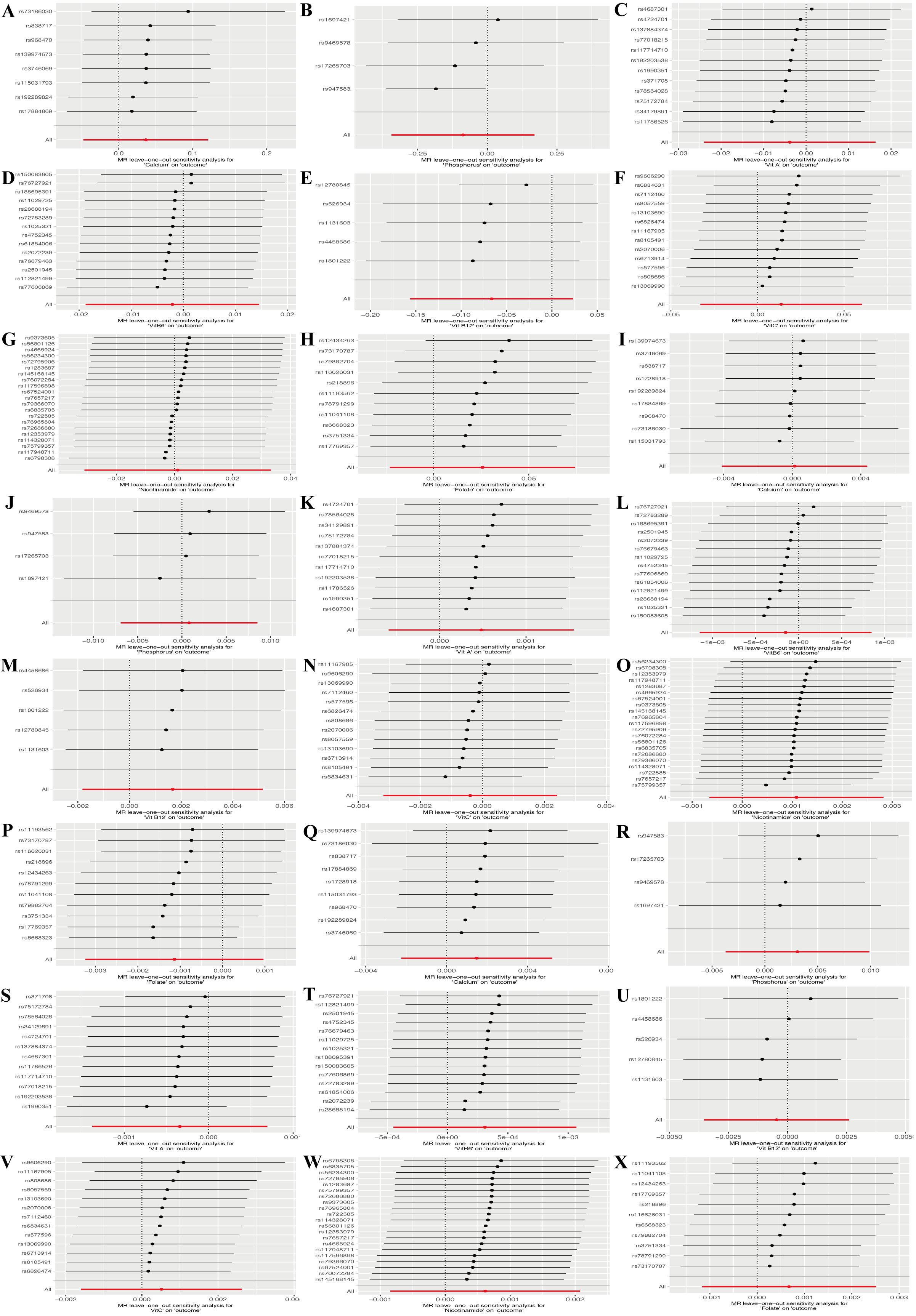


**Figure S4.** Leave-one-out sensitivity analysis of the discovery set. (A-H) Exposures (Ca, P, vit A, B6, B12, C, nicotinamide, folate) and gestational diabetes mellitus; (I-P) Exposures and gestational hypertension; (Q-X) Exposures and spontaneous abortion.


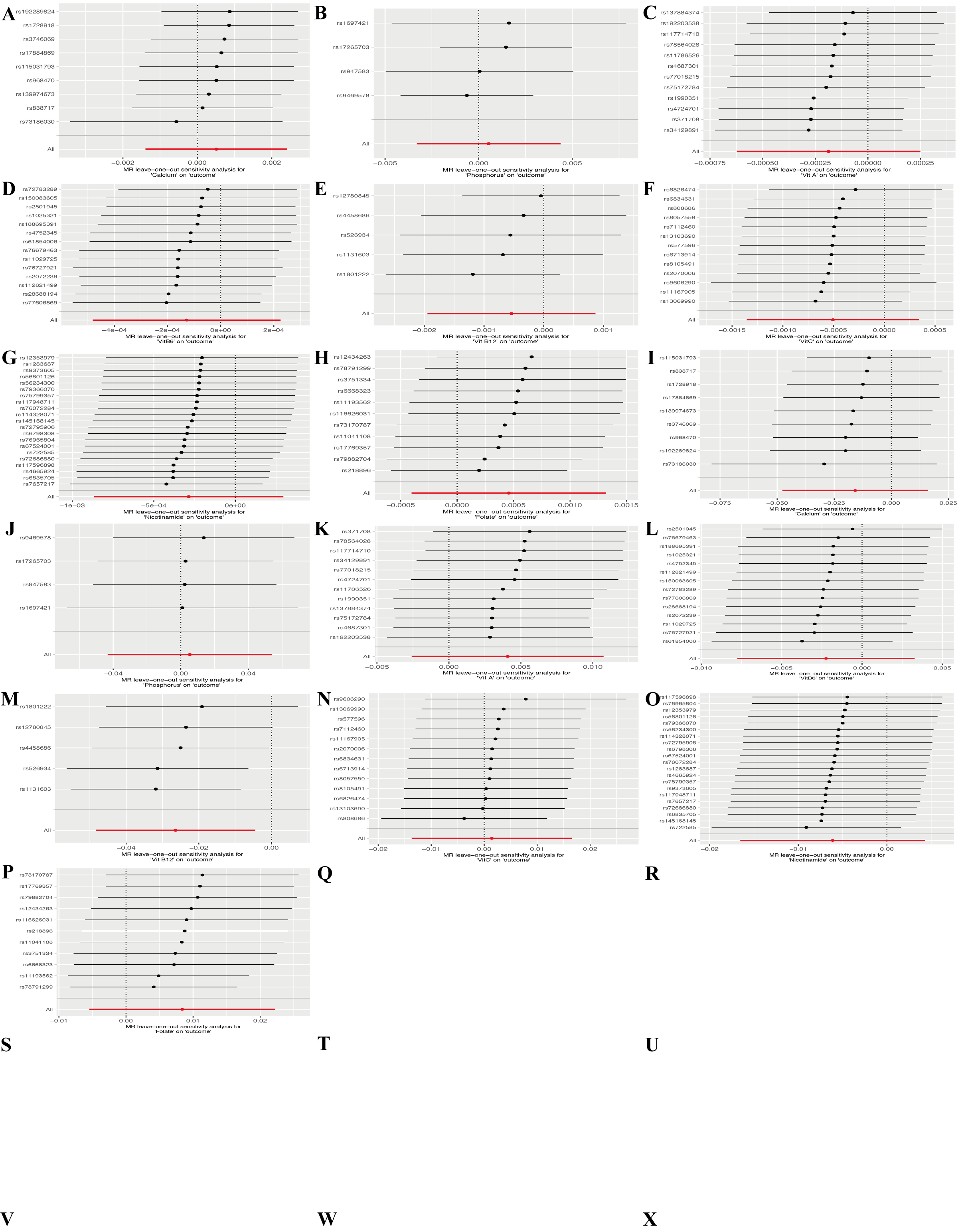


**Figure S5.** Leave-one-out sensitivity analysis of the discovery set. (A-H) Exposures (Ca, P, vit A, B6, B12, C, nicotinamide, folate) and preterm birth; (I-P) Exposures and still birth.


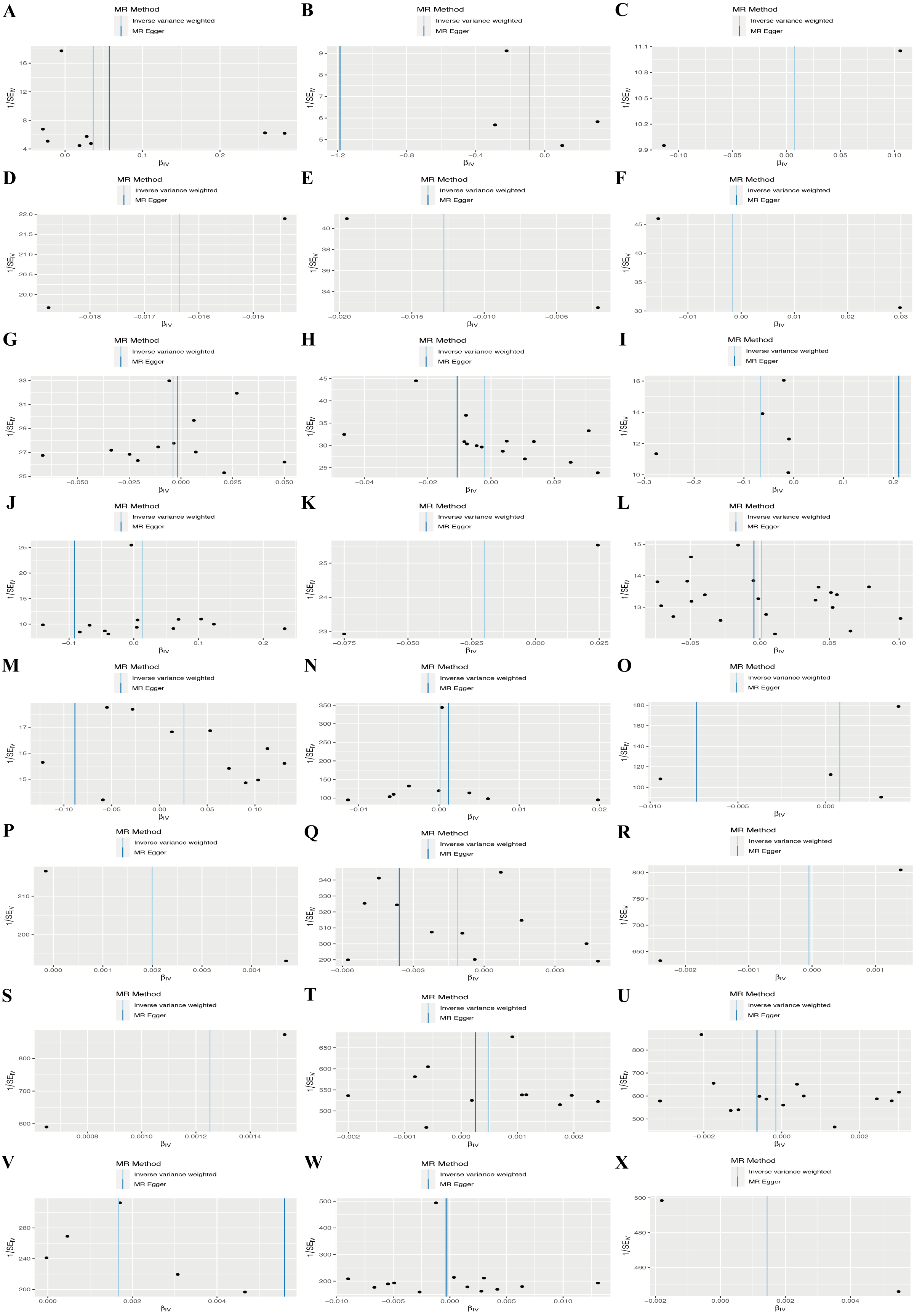


**Figure S6.** Funnel plots of the discovery set. (A-M) Exposures (Ca, P, Mg, Fe, Zn, Cu, vit A, B6, B12, C, D, nicotinamide, folate) and gestational diabetes mellitus; (N-X) Exposures (Ca, P, Mg, folate, Zn, Cu, vit A, B6, B12, C, D) and gestational hypertension.


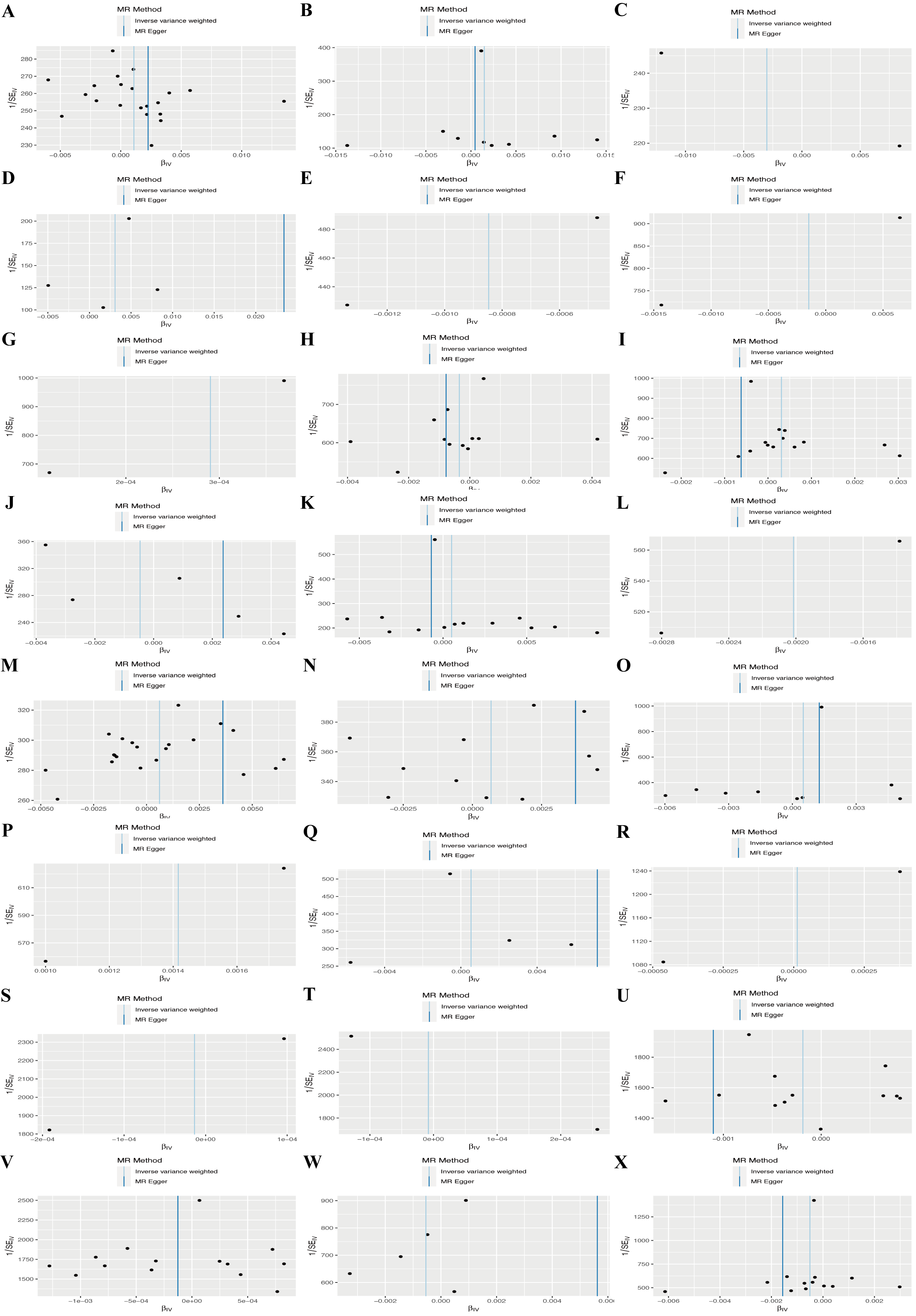


**Figure S7.** Funnel plots of the discovery set. (A) Exposures (Nicotinamide) and gestational hypertension; (B-N) Exposures (Ca, P, Mg, Fe, Zn, Cu, vit A, B6, B12, C, D, nicotinamide, folate) and spontaneous abortion; (O-X) Exposures (Ca, P, Mg, Fe, Zn, Cu, vit A, B6, B12, C) and preterm birth.


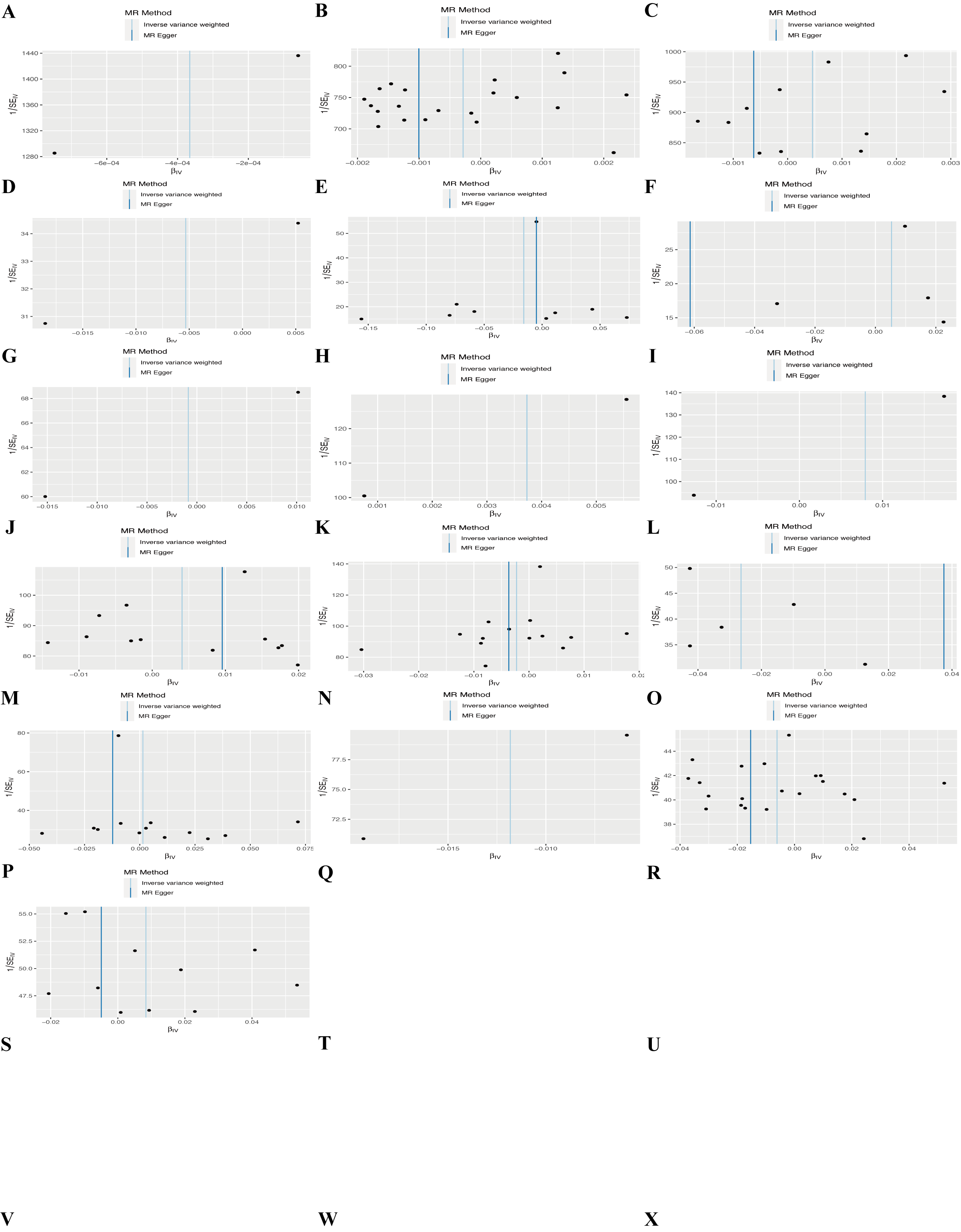


**Figure S8.** Funnel plots of the discovery set. (A-C) Exposures (Vit D, nicotinamide, folate) and preterm birth; (D-P) Exposures (Ca, P, Mg, Fe, Zn, Cu, vit A, B6, B12, C, D, nicotinamide, folate) and still birth.
